# Human Milk Oligosaccharides Are Associated with Lactation Stage and Lewis Phenotype in a Chinese Population

**DOI:** 10.3390/nu15061408

**Published:** 2023-03-15

**Authors:** Xiangnan Ren, Jingyu Yan, Ye Bi, Paul William Shuttleworth, Ye Wang, Shan Jiang, Jie Wang, Yifan Duan, Jianqiang Lai, Zhenyu Yang

**Affiliations:** 1 National Institute for Nutrition and Health, Chinese Center for Disease Control and Prevention, Beijing 100050, China; 2 Key Laboratory of Trace Element Nutrition of National Health Commission, Beijing 100050, China; 3 Key Laboratory of Human Milk Science, Chinese Center for Disease Control and Prevention, Beijing 100050, China; 4 Dalian Institute of Chemical Physics, Chinese Academy of Sciences, Key Laboratory of Separation Science for Analytical Chemistry, Dalian 116023, China; 5 Department of General Surgery, Tameside and Glossop Integrated Care NHS Foundation Trust, Ashton-under-Lyne OL69RW, UK

**Keywords:** human milk, oligosaccharides, lactation stage, secretor gene status, Lewis blood type

## Abstract

Background: Human milk oligosaccharides (HMOs) are the third most abundant component of human milk. Various factors may affect the concentration of HMOs, such as the lactation period, Lewis blood type, and the maternal secretor gene status. Objectives: The purpose of this study is to investigate factors associated with HMO concentrations in Chinese populations. Methods: A sub-sample of 481 was randomly selected from a large cross-sectional study in China (*n* = 6481) conducted in eight provinces (Beijing, Heilongjiang, Shanghai, Yunnan, Gansu, Guangdong, Zhejiang, and Shandong) between 2011 and 2013. HMO concentrations were determined by a high-throughput UPLC-MRM method. Various factors were collected through face-to-face interviews. Anthropometric measurement was conducted by trained staff. Results: Median total HMO concentration was 13.6 g/L, 10.7 g/L, and 6.0 g/L for colostrum, transitional milk, and mature milk, respectively. HMO concentration decreased significantly as the lactation period increased (*p <* 0.0001). There were significant differences of average total HMO concentration between secretor mothers and non-secretor mothers (secretor 11.3 g/L vs. non-secretor 5.8 g/L, *p <* 0.0001). There were significant differences of average total HMO concentrations among three Lewis blood types (*p =* 0.003). Comparing with the concentration of total oligosaccharides of Le+ (a−b+), average of total oligosaccharides concentrations increased by 3.9 (Le+ (a+b−), *p =* 0.004) and 1.1 g/L (Le− (a−b−), *p =* 0.049). The volume of breast milk expressed and the province the mother came from affected the concentration of total oligosaccharides (all *p <* 0.0001). Maternal BMI (*p* = 0.151), age (*p* = 0.630), prematurity (*p* = 0.850), mode of delivery (*p* = 0.486), infants’ gender (*p* = 0.685), maternal education level (*p* = 0.989), maternal occupation (*p* = 0.568), maternal allergic history (*p* = 0.370), maternal anemia (*p* = 0.625), pregnancy-induced hypertension (*p* = 0.739), gestational diabetes (*p* = 0.514), and parity (*p* = 0.098) were not significantly correlated with the concentration of milk oligosaccharides. The concentrations of 2′-fucosyllactose (2′-FL), lacto-N-neotetraose (LNnT), sialyllacto-N-tetraose c (LSTc), lacto-N-fucopentaose I (LNFP-I), disialylated lacto-N-tetraose (DSLNT), difucosyl-para-lacto-N-neohexaose (DFpLNnH), difucosyl-lacto-N-hexaose (DFLNH[a]), and 3-sialyllactose (3′-SL) showed a gradual downward trend, while the concentration of 3-fucosyllactose (3-FL) showed a gradual upward trend among three lactation stages (*p <* 0.05). Conclusions: The concentration of HMOs changes throughout lactation, and it varies between different HMOs. HMO concentrations differed between lactation stage, maternal secretor gene status, Lewis blood type, volume of breast milk expressed, and the province the mother came from. Prematurity, mode of delivery, parity, infants’ gender, and maternal characteristics did not affect the HMO concentration. Geographical region may be not associated with HMOs concentration in human milk. There may be a mechanism for co-regulation of the secretion of some of the oligosaccharides such as 2′FL vs. 3FL, 2′FL vs. LNnT, and lacto-N-tetraose (LNT).

## 1. Introduction

Human milk oligosaccharides (HMOs) are complex glycans that are indigestible by infants, and are the third most abundant component in human milk [1]. The mean total concentration of HMOs ranges from 5 g/L to 20 g/L, and there are different types and lower quantities of oligosaccharides in bovine and goat milk compared with human milk [1,2,3]. Recently, researchers have suggested that HMOs play an important role in biological functions, such as acting as prebiotics, antiadhesives preventing pathogen adhesion, and affecting immune modulation [2,3,4,5,6]. HMOs may play an important role in promoting a healthy, micro-ecological environment, nourishing health-promoting bacteria in an infant’s gastrointestinal tract [4,7,8]. HMOs could also play a role in modulating cognitive functions [9]. Several interventional trials have supported these potential roles, suggesting that HMOs could not only reduce the incidence of neonatal necrotizing enterocolitis, but could also be involved in the development of immune function and brain development [9,10]. 

HMO concentrations are affected by several factors, such as the lactation period, Lewis blood type and secretor gene type, regional characteristics, infants’ health and prematurity, maternal disease, and parity. Fucosylated HMO production is controlled by α-1-2, α-1-3 and α-1-4 fucosyltransferase [11]. The expression of α-1-2, fucosyltransferase is closely related to FUT2 (Se gene), and the expression of α-1-3, α-1-4 fucosyltransferase is closely related to FUT3 (Lewis gene) [12,13,14,15,16]. HMOs have been investigated in various countries, with a limited sample size ranging from 9 to 450 samples [1,17,18,19,20,21,22,23,24]. Numerous papers have shown that lactation time is the most significant factor affecting the concentration of HMOs, and the total concentration of HMO in colostrum is higher than those in mature milk [1,17,24]. Lewis blood type and maternal genetics may affect the HMO concentrations [17,19,23,24]. The relative concentrations of the various types of HMO have also been shown to vary among populations across different countries, suggesting that geographical location may be one of the potential influencing factors affecting their relative abundance [1]. In pregnancies carried to full term, the concentration of HMO secreted by mothers has been shown to be lower than in babies delivered prematurely [24]. 

However, sample sizes in the previously published evidence are small, and the type of available studies are limited. Subject populations generally came from the same region or the same living environment, which might limit the generalization of the results to a large population. Additionally, there were no standardized methods in analyzing, and the comparability of values in different studies still needs to be explored. Changes in the concentration of HMO throughout lactation in different living environments, and in different parts of China, have not been reported. The objectives of this study were to analyze dynamic changes of HMO concentration during different lactation periods (colostrum, transitional milk, mature milk) from a cross-sectional survey in China, and to explore the potential influencing factors that may affect concentrations of HMO. 

## 2. Methods 

### 2.1. Study Design

The study design has been described previously in a paper reporting as part of building a regional breast milk composition database in China [25]. This study reports a sub sample of 481 samples from different women, stratified and randomly selected from 6481 human milk samples, from multiple sites including eight provinces (Beijing, Heilongjiang, Shanghai, Yunnan, Gansu, Guangdong, Zhejiang, and Shandong). It includes not only urban and rural areas, but also inland and coastal areas. The healthy lactating mothers were at different stages of lactation (0~340 days postpartum). Colostrum was 0~6 days postpartum, transitional milk was 7~14 days postpartum, and mature milk was 15~340 days postpartum. 

Ethical approval was given by the ethics committee of the National Institute for Nutrition and Health, China CDC. Written informed consent was obtained from all participants.

### 2.2. Human Milk Sample Collection

Human milk samples were collected in a room without direct sunlight exposure. One full breast was emptied by a portable electric breast pump (HNR/X-2108Z, Shantou, Guangdong, China) into a feeding bottle in the morning (9:00~11:00 a.m.). After gently agitating the bottle for ~10 times, the samples were divided into 15 mL centrifuge tubes and were stored at −20 °C in a freezer. The frozen samples were shipped to a central laboratory at the National Institute for Nutrition and Health, CCDC, in Beijing and stored in a −80 °C freezer until analysis. The cold chain was preserved throughout.

### 2.3. Analysis of Human Milk Oligosaccharides

Human milk oligosaccharide (HMO) content was determined using a recently published high-throughput UPLC-MRM method [26]. An ACQUITY Ultraperformance system (Waters, Milford, MA, USA) coupled to a Xevo TQ-XS triple quadrupole mass spectrometer (Waters) was used for analysis. The mobile phase solvents consisted of acetonitrile and water with ammonium acetate as the additive. ESI-MS detection was in the negative-ion mode, and collision-induced dissociation tandem MS (CID-MS/MS) was carried out using multi-reaction monitoring (MRM) for both sequence assignment and quantitation. The samples were centrifuged at 8000× *g* rpm for 6 min at 4 °C in order to remove the fat component. After removal of the top lipid layer, the sample was diluted by a factor of 15 using ultrapure water. For analysis, an aliquot (100 μL) of the sample solution was taken out and 2 volumes of ethanol added. The mixture was then centrifuged at 8000× *g* rpm for 6 min at 4 °C. The supernatant was 1:1 diluted with 50% ACN in H_2_O (*v*/*v*). The overall dilution of the milk sample in various solvent was 1:90. The quantitation calibration curves of oligosaccharides were used, and each standard curve covered 8 concentration points. To make the standard concentrations closer to those of the real samples, we divided the oligosaccharide standards into high- and low-content groups and set different concentration ranges. In order to ensure a good parallelism in terms of MRM detection sensitivity and the chromatographic resolution (e.g., peak shape and retention time) for the large number of sample analyses, the mixed standard solution was used for quality control by injection between every 10 samples analyzed. 

### 2.4. Maternal Secretor Status and Geographical Factors 

The subjects’ secretor status and Lewis blood type was determined by the presence of specific oligosaccharide and ion fragments. The presence of the product ion *m*/*z* 325 from 2′-fucosyllactose (2′-FL), Lactodifucotetraose (LDFT), lacto-*N*-fucopentaose I (LNFP-I), and lacto-*N*-neo-difucohexaose I (LNnDFH-I) can be used as an indicator of secretor’s status, while lacto-*N*-fucopentaose II (LNFP-II) with the fragment ion *m*/*z* 348 can be used as an indicator of Lewis blood-group phenotype [26,27]. Study sites were divided into rural and urban areas or were classified into coastal and inland areas based on geographic features.

We used the sum of the concentrations of lacto-N-difucohexaose I (LNDFH-I) and LNnDFH-I for all the data analysis because the concentration of LNnDFH-I was so low that it was not even detected in some milk samples. These two oligosaccharides shared the common characteristics of only being found in secretors milk samples; therefore, we used the sum of two oligosaccharides instead of the individual concentrations.

### 2.5. Statistical Analysis

Descriptive statistics were calculated for both continuous variables and categorical variables. All continuous variables were tested for normality. The data were expressed as mean ± SD and median. The median of concentrations of oligosaccharides was used in the data presented in chart form. The concentration of some oligosaccharides were heavily skewed, and therefore the natural log was used. The logarithmic values were used for statistical analysis. Nonparametric tests were used if the logarithmic data were still skewed. Analysis of variance (ANOVA) was used to analyze the effects of different lactating stages and blood type. Independent sample t-test was used to study the relationship between delivery time (term vs. preterm), between living environments (rural areas vs. urban areas), and between geographical location (coastal areas vs. inland areas). The Tukey–Kramer test was used for adjusting multiple comparisons if there were statistical significance for overall effects. The general linear model was selected to analyze the relationship between human milk oligosaccharides and influencing factors. Selecting the significant and possible influencing factors to enter the multiple regression model. Differences were considered significant at *p* < 0.05. Principal component analysis (PCA) was used to obtain an overview of variations among oligosaccharides at different lactation stages. All analyses were conducted with SAS 9.4 (SAS Inc., Cary, NC, USA) or Origin software 2019 (Originlab, Northampton, MA, USA).

## 3. Results

### 3.1. Characteristics of Study Subjects

The characteristics of the subjects are shown in Table 1. The mean maternal age of lactating women was 26.8 years, and mean pre-pregnancy BMI of all mothers was 20.8 kg/m^2^; 54.3% of the studied infants were boys. The proportion of preterm infants was 4.4%. Mean birth weights of term and preterm infants were 3491.3 g, and 2845.2 g, respectively. Colostrum, transitional milk, and mature milk samples each accounted for about one third of the total samples. The proportions of rural and urban areas were 36.8% and 63.2%, respectively. The proportions of coastal and inland areas were 31.8% and 68.2%, respectively. 

### 3.2. Concentrations of Human Milk Oligosaccharides during Different Lactation Periods

**Figure 1:**
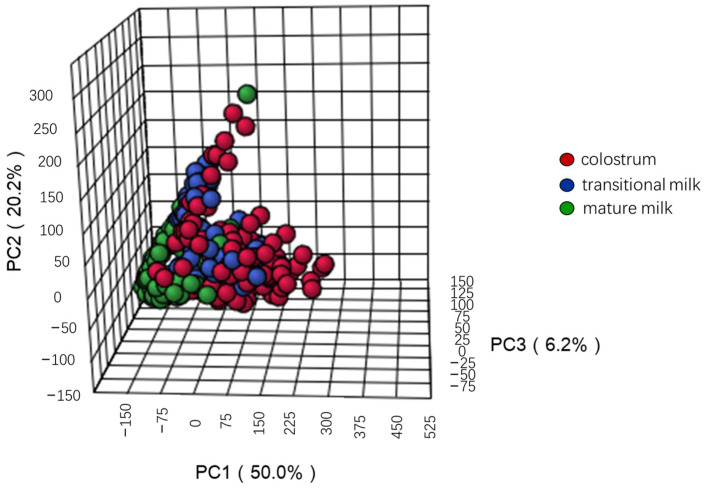
PCA of oligosaccharides at different lactation stages.

**Figure 2:**
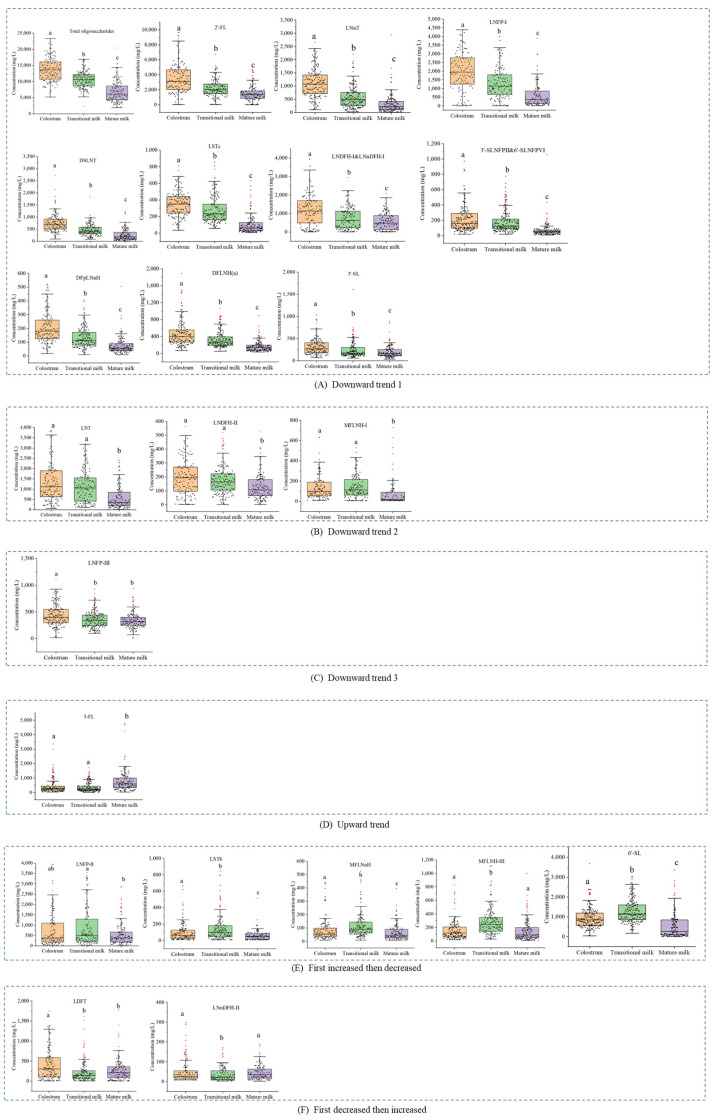
Box diagram of oligosaccharides in three lactation stages. Data are shown as median and quartiles. Different small letters indicate a significant difference (*p* < 0.05). Brackets with numbers indicate the figure number.

Principal component analysis (PCA) of HMOs showed a spectral separation among different lactation stages, indicating significant differences of concentrations of oligosaccharides (Figure 1). The first principal component (PC1) with PC2 and PC3 described 76.4% of the variation contained in the concentrations of HMO from different lactation stages. The dispersion of within-particular groups can be observed on the PCA score plot and indicated a small variance in HMO concentrations among mature milk samples. There were significant differences in the concentrations of 23 oligosaccharides among three lactation stages based on ANOVA (*p* < 0.05). The box diagrams of oligosaccharides during three lactation stages are shown in Figure 2; lower case letters represent significant differences. Median total HMO concentration was 13.6 g/L, 10.7 g/L, and 6.0 g/L for colostrum, transitional milk, and mature milk, respectively, and decreased significantly the longer the interval since birth (*p* < 0.0001). The concentrations in colostrum were the highest and the concentrations in mature milk were the lowest, and showed a gradual downward trend in concentration for the following 14 oligosaccharides: 2′-FL, lacto-N-neotetraose (LNnT), sialyllacto-N-tetraose c (LSTc), LNFP-I, disialylated LNT (DSLNT), difucosyl-para-lacto-N-neohexaose (DFpLNnH), difucosyl-lacto-N-hexaose (DFLNH[a]), 3-sialyllactose (3′-SL). Through pairwise comparison, there were significant differences in HMO concentrations between any two groups (colostrum, transitional milk, mature milk) for these oligosaccharides (*p* < 0.05). The concentration of 3-fucosyllactose (3-FL) showed a gradual upward trend during lactation stages, and the concentrations in mature milk were higher than in colostrum and transitional milk (*p* < 0.05). The concentration of lacto-difucotetraose (LDFT) first decreased and then increased, and the concentration in colostrum was higher than in transitional milk, or mature milk (*p* < 0.05). The concentrations of other oligosaccharides also first increased then decreased, including sialyllacto-*N*-tetraose b (LSTb), 6-Sialyllactose (6′-SL), LNFP-II, monofucosyl-lacto-*N*-neohexaose (MFLNnH), and monofucosyl-lacto-N-hexaose III (MFLNH-III). The concentrations of oligosaccharides at different lactation stages are shown in Table S1. 

**Figure 3:**
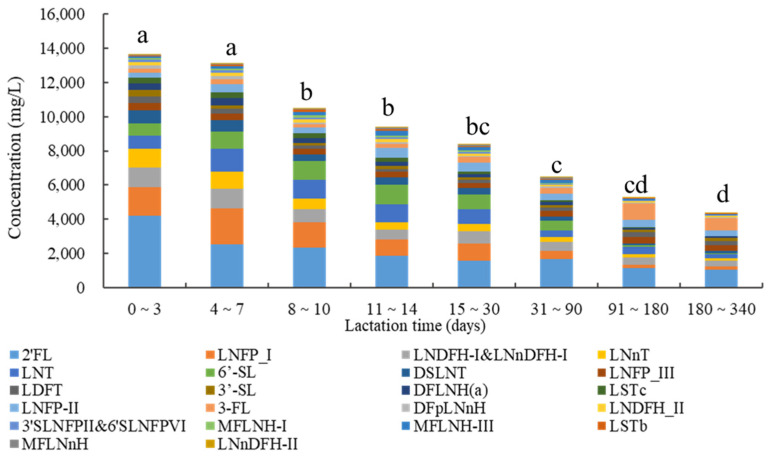
Concentrations of total oligosaccharides at different lactation times. Data are shown as median. Small letters indicate a significant difference (*p* < 0.05).

Concentrations of HMOs during different lactation periods are shown in Figure 3. The concentrations of total oligosaccharides showed a downward trend as lactation duration increased, with negative linear correlation between the total concentration of oligosaccharides and lactation time (r = −0.592, *p* < 0.0001). Comparing with the total concentration of oligosaccharides on lactation 0~3 day, the median of total oligosaccharides concentrations decreased by 2.5 g/L (day 8~10), 3.2 g/L (day 11~14), 4.2 g/L (day 15~30), 6.1 g/L (day 31~90), 8.3 g/L (day 91~180), and 9.0 g/L (day 181~340), respectively (all *p* < 0.01). There were no significant differences of total oligosaccharides between day 0~3 and day 4~7 (*p* = 0.713). Some specific oligosaccharides showed a downward trend in relation to lactation duration, such as 2′-FL, 3′-SL, LNnT, DSLNT, LSTc, DFLNH(a), and DFpLNnH. The concentrations of lacto-*N*-tetraose (LNT), LNFP-I, 6′-SL, and LSTb were highest during lactation day 4~7, day 4~7, day 11~14, and day 8~10, respectively. 3-FL showed an upward trend as the duration of lactation increased. 

### 3.3. HMOs in Different Geographical Factors and Preterm Infants

**Figure 4:**
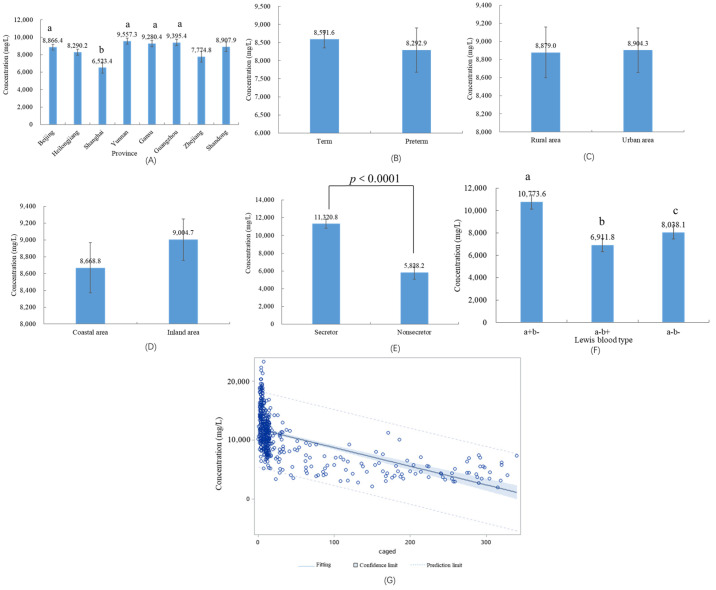
Concentrations of total oligosaccharides in different influencing factors: (**A**) different provinces; (**B**) preterm and term; (**C**) rural areas and urban areas; (**D**) coastal areas and inland areas; (**E**) active secretor gene and inactive secretor gene; (**F**) Lewis blood type; (**G**) lactation days. Data are shown as mean ± SE. Small letters indicate significant differences (*p* < 0.05). Brackets with numbers indicate figure number.

The province the subjects came from could affect the concentration of total oligosaccharides (*p <* 0.0001). Comparing the concentration of total oligosaccharides from Shanghai province, the average of total oligosaccharides concentrations increased by 2.3 g/L (Beijing), 3.0 g/L (Yunnan), 2.8 g/L (Gansu), and 2.9 g/L (Guangzhou), respectively (all *p <* 0.05) (Figure 4A). There were no significant differences between rural areas and urban areas (*p* = 0.922) or between coastal areas and inland areas (*p* = 0.244) (Figure 4C,D). There were no significant differences of total oligosaccharides secreted, between preterm milk and full-term milk (*p* = 0.618) (Figure 4B).

### 3.4. HMOs in Different Maternal Secretor Status and Lewis Blood Type

**Figure 5:**
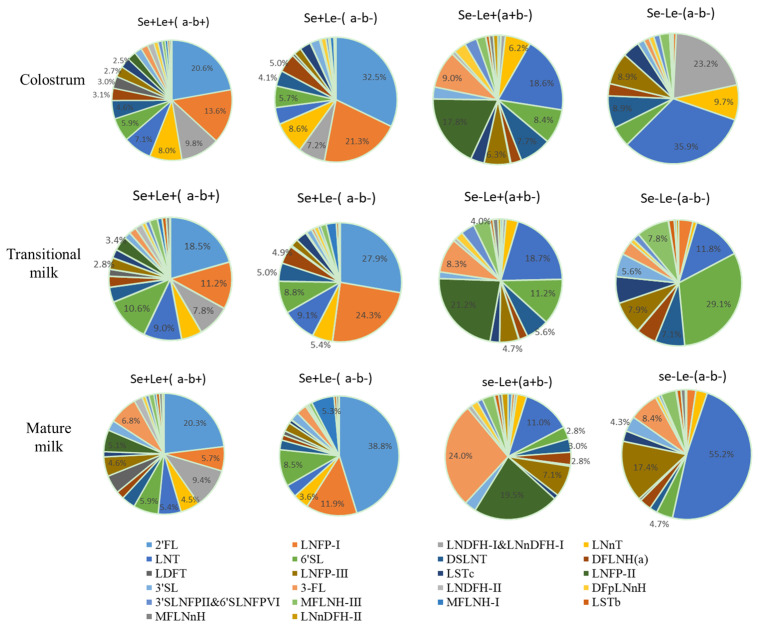
Percentage of human milk oligosaccharides in different blood groups, three lactation stages.

The percentages of secretor and non-secretor mothers were 75.4%, and 24.6%, respectively. There were significant differences in the average of the total amount of HMOs secreted between secretor mothers and non-secretor mothers (secretor (11.3 g/L) vs. non-secretor (5.8 g/L), *p* < 0.0001) (Figure 4E). Concentrations of 2′-FL, LDFT, LNFP-I, and MFLNH-I particularly, were far lower in milk from non-secretor mothers than those from secretor mothers (*p* < 0.0001). The concentrations of 3-FL, LNT, LNFP-II, lacto-*N*-neo-difucohexaose II (LNnDFH-II), and monofucosyl-lacto-*N*-hexaose III (MFLNH-III) were approximately 2~5 times higher in milk from non-secretor mothers than those from secretor mothers (*p* < 0.0001). The percentages of Le+ (a+b−), Le+ (a−b+), and Le− (a−b−) were 22.9%, 68.6%, and 8.5%, respectively. The Lewis blood type also affected the concentration of total oligosaccharides (*p* = 0.003). Comparing the concentration of total oligosaccharides of Le+ (a−b+), the average total oligosaccharides concentrations increased by 3.9 (Le+ (a+b−), *p =* 0.004) and 1.1 g/L (Le− (a−b−), *p =* 0.049) (Figure 4F). Lewis phenotype takes into account both maternal secretor gene status and Lewis blood group. There is a significant difference between the percentage of different oligosaccharides in different Lewis phenotypes. (Figure 5). The percentage of 2′-FL was highest (above 18.7%) compared with other oligosaccharides for Se+Le+ (a−b+) blood type. The percentage of 2′-FL was the highest specific oligosaccharide for Se+Le− (a−b−) blood type (colostrum: 33.8%, transitional milk: 23.9%, mature milk: 38.8%). The percentages of LNT, LNFP-II, and 3-FL were higher than other oligosaccharides for Se−Le+ (a+b−) blood type: 18.8%, 17.6%, 8.1% in colostrum; 18.7%, 21.2%, 8.3% in transitional milk; 11.0%, 19.5%, 24.0% in mature milk. For colostrum, percentages of LNT (39.2%) were higher than other oligosaccharides for Se−Le− (a−b−) blood type. For transitional milk, percentages of 6′-SL (29.1%) and LNT (11.8%) were higher than other oligosaccharides for Se−Le− (a−b−) blood type. For mature milk, the percentages of LNT (55.2%) and LNFP-III (17.4%) were higher than other oligosaccharides for Se−Le− (a−b−) blood type. The percentage of HMOs in different blood groups in the three lactation stages are shown in Table S2. 

### 3.5. Influencing Factors on the Concentrations of HMOs

According to a multiple regression model analysis, the total concentration of oligosaccharides was closely associated with lactation duration, maternal secretor gene type, Lewis blood type, the volume of breast milk expressed, and the province the mother came from. R^2^ was 0.6154. After correcting for other factors, Figure 4G shows a scatter plot charting the total concentration of HMOs, versus the number of days the mother has been lactating. The line of best fit shows a negative correlation, with total HMO concentration decreasing with time. Maternal BMI (*p* = 0.151), age (*p* = 0.630), prematurity (*p* = 0.850), mode of delivery (*p* = 0.486), infants’ gender (*p* = 0.685), maternal education level (*p* = 0.989), maternal occupation (*p* = 0.568), maternal allergic history (*p* = 0.370), maternal anemia (*p* = 0.625), pregnancy-induced hypertension (*p* = 0.739), gestational diabetes (*p* = 0.514), and parity (*p* = 0.098) were not significantly correlated with the concentration of milk oligosaccharides. 

### 3.6. Correlation of Human Milk Oligosaccharides

**Figure 6:**
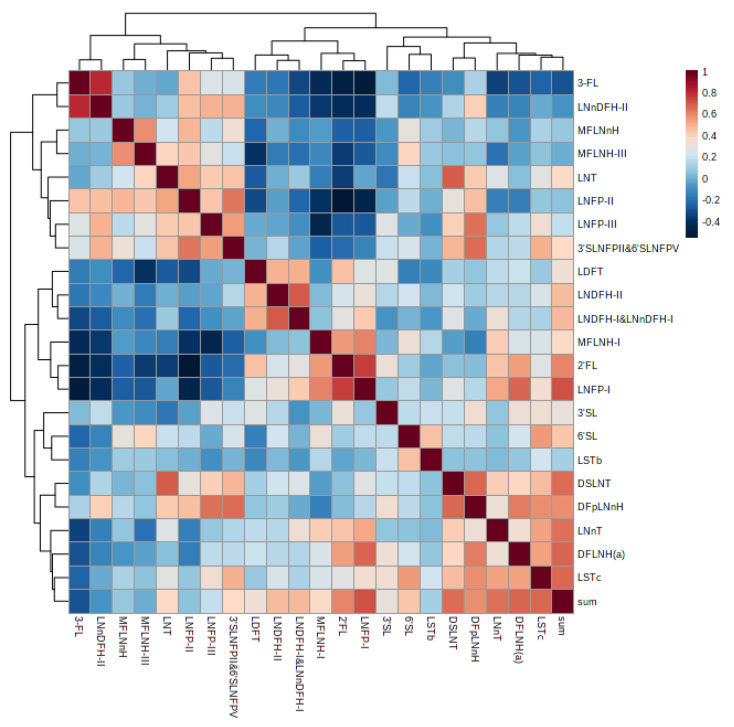
Correlation of human milk oligosaccharides.

The correlations between the concentrations of the various human milk oligosaccharides are shown in Figure 6. The correlations were the absolute abundance of these specific HMOs over the period of lactation. 3-FL had a strong positive correlation with LNnDFH-II (r = 0.804, *p* < 0.0001). 3-FL had a strong negative correlation with MFLNH-I (r = −0.623, *p* < 0.0001), 2′-FL (r = −0.514, *p* < 0.0001), and LNFP-I (r = −0.676, *p* < 0.0001). LNnDFH-II had a strong negative correlation with MFLNH-I (r = −0.625, *p* < 0.0001), 2′-FL (r = −0.413, *p* < 0.0001), and LNFP-I (r = −0.503, *p* < 0.0001). MFLNH-III had a strong negative correlation with LDFT (r = −0.447, *p* < 0.0001). LNFP-II had a strong negative correlation with 2′-FL (r = −0.467, *p* < 0.0001). LNFP- III had a strong negative correlation with MFLNH-I (r = −0.484, *p* < 0.0001). 2′-FL had a strong positive correlation with LNFP-I (r = 0.699, *p* < 0.0001). LNT had a strong positive correlation with DSLNT (r = 0.692, *p* < 0.0001). Detailed information of the correlation of human milk oligosaccharides is shown in Table S3.

## 4. Discussion

Our study analyzed the concentrations of 24 different HMOs in 481 human milk samples, in a Chinese population. We assessed for the influence of varying factors, including prematurity, geographical region, and others. The median of the total HMO concentration was 13.6 g/L, 10.7 g/L, and 6.0 g/L for colostrum, transitional milk, and mature milk, respectively, and decreased significantly with the extension of lactation days. There were significant differences of total human milk oligosaccharides between secretor mothers and non-secretor mothers. 

This study demonstrates that the concentrations of HMO changed dynamically the longer the mother had been lactating. The concentrations of total HMO gradually decreased as lactation extended, and the same trend was discovered in 2′-FL, 3′-SL, LNnT, DSLNT, and LSTc; this is consistent with previous studies [3,17,28]. 3-FL demonstrated a dynamic increase in concentration, which is also consistent with previous evidence [3,17,24,28,29]. The trends in the changes of the amount of LDFT, LSTb, 6′-SL, LNFP-I, and LNT secreted were also consistent with the published literature. Gabrielli et al. [24] have suggested that during the first month of lactation, decreases in total oligosaccharide concentrations occur for Se+Le+ groups and Se−Le− groups (14.7% and 21.5%, respectively), whereas minimal variations were present in Se−Le+ groups and Se+Le− groups. Total HMO concentrations decreased in four groups as the lactation period extended in our study. Their paper was a cohort study, while our study was a cross-sectional study. The sample size of the Se+Le− group (*n* = 7) and Se−Le− group (*n* = 7) was relatively small. Concentrations of DFLNH(a), LNFP-II, DFpLNnH, MFLNH-III, MFLNH-I, MFLNnH, and LNnDFH-II have not previously been reported in Chinese populations, and we present them for the first time. The concentrations of LNFP-II, MFLNnH, and MFLNH-III first increased, then decreased; the concentration of LNnDFH-II first decreased, then increased. The concentrations of DFLNH(a) and DFpLNnH showed a downward trend, which has not previously been reported with a clear trend in prior publications. 

The combined total concentrations of HMO was similar in colostrum, transitional milk, and mature milk from mothers of term and preterm infants, which is consistent with previous studies [19]. A slightly higher concentration of HMO was found in urban areas compared with rural areas, and a slightly higher concentration of HMO was found in coastal area compared with inland areas, but these were not statistically significant, *p* > 0.05. The geographic region and ethnic group the mother comes from significantly affects the secretion of HMO. McGuire et al. [1] suggested normal HMO concentrations and profiles vary geographically. Li et al. [26] indicated that HMO concentration of the Tibetans was 56% higher than the concentration of Zhuang, and the concentration of Han (6.3 g/L) was close to the average value (5.6 g/L) of all samples, which was within the scope of our research results. This result is the average of all data during different lactation periods (colostrum, transitional, and mature milk), with a mean lactation value of 143 days, with the earliest at day 6 and the longest at day 347. The total HMO concentrations in different lactation periods was reported instead of giving the average value of all data in our research because lactation stage was the important factor. Different ethnic groups may have different living environmental conditions and dietary habits that could explain these differences. The Han ethnic group was the largest, and the results of the Han ethnic group were close to the average level, therefore this article analyzes the oligosaccharide data of the Han population in detail. This can avoid the influence of too many confounding factors. However, living environments and geographical location are intimately related, and both may have some impact affecting the results. Therefore, these factors were analyzed using a multiple regression model.

This study revealed that the total HMO concentrations, and the concentration of an individual HMO, showed large differences depending on the Lewis blood type (active or inactive FUT3 gene) and maternal secretor gene status (active or inactive FUT2 gene) [12,13,14,15,16]. The different expression of these two genes affects the Lewis blood group and maternal secretor status. The percentages of secretor (Se+) and non-secretor (Se−) status in this study were 75.4%, and 24.6%, respectively, which were similar to numerous studies [30,31]. The secretor (Se) gene encoded for the FUT2 is necessary for the synthesis of 2′FL and other α1-2-fucosylated HMOs [13]. There was an abundance of α1-2-fucosylated HMOs in milk from secretor mothers, such as 2′FL, LNFP I, and LDFT. By contrast, in non-secretors who lack the FUT2 enzyme, the concentrations of α1-2-fucosylated HMOs were only minimal, or not present. The absence of α-1-2-linked fucosylated oligosaccharides in milk from non-secretor mothers helps explain the lower total concentration of HMOs. 2′FL was highest in milk from secretor mothers. The percentages of 2′FL in colostrum, transitional milk, and mature milk from secretor mothers were 53.1%, 46.4%, and 58.8%, respectively, in this study. Because of high amounts secreted in breast milk, and the relative importance of 2′FL, it is available in some commercial infant formulas [32]. The USA’s FDA and the European Food Safety Authority (EFSA) considers that 2′FL is generally safe for infants under one year of age. 2′FL may improve infants gut microbiota composition, inhibit bacterial infection via antiadhesion mechanism, modulate the intestinal epithelial cell response, and regulate immunity [32,33]. It has also been shown to influence cognitive function and improve learning and memory in rodents [33,34,35,36,37]. The concentrations of LNT were the highest in milk from non-secretor mothers, and percentages in colostrum, transitional milk, and mature milk were 54.5%, 30.5%, and 65.8%, respectively. Sprenger et al. [22] have suggested that LNnT and LNT are ‘co-regulated’ with the FUT2-dependent 2′FL concentration. LNT concentration was negatively correlated with the amount of 2′FL, and LNnT concentration was positively correlated, which is consistent with the published literature [22]. It has been suggested that LNT and LNnT protect against important systemic infections of the newborn [13]. The secretion of 3-FL shows a gradual upward trend during lactation stages. 3-FL concentration was negatively correlated with the amount of 2′FL, which is consistent with previous studies [22]. Austin et al. [17] demonstrated the strong negative correlation between 2′-FL and 3-FL concentration, suggesting there is a possible mechanism of co-regulation. The EFSA Panel on Nutrition have evaluated the safety of 3-FL as a novel food and suggested 3-FL was safe under the proposed conditions of use, including the use as a food supplement [38]. A few studies have been conducted on the functionalities of 3-FL. 2′-FL and 3-FL have been reported to play an important role in the establishment of a healthy gut microbiome by selectively stimulating the growth of beneficial bacterium and suppressing the growth of harmful bacteria [39]. More research into the prebiotic effects of 3-FL is needed to confirm this. 

Lewis phenotype takes into account both maternal secretor gene status and Lewis blood group, and has been shown to play a key role in determining HMO secretion. They can be combined to generate an *SeLe* phenotype. The percentages of Lewis blood group (a−b+), (a+b−), and (a−b−) in this study were 68.6%, 22.9%, and 8.5%, respectively. The FUT3 gene expression produces α-1-3/4-L-fucosyltransferase, which controls the production of α-1-3/4-L-fucosylated oligosaccharides. The product of the FUT2 gene expression is α-1-2-L-fucosyltransferase, which controls the production of α-1-2-L-fucosylated oligosaccharides. 4 *SeLe* phenotype are controlled by FUT2 and FUT3 jointly [29,31]. Considering the maternal secretor status and Lewis blood group, the percentages of Se+Le+ (a−b+), Se+Le− (a−b−), Se−Le+ (a+b−), and Se−Le− (a−b−) were 68.1%, 7.3%, 22.7%, and 1.9%, respectively. The main types of maternal milk phenotype were Se+Le+ (a−b+) and Se−Le+ (a+b−), and the incidence of Se−Le− (a−b−) was less than 2%, which is consistent with the published evidence [24,31]. Overall, the proportion of these blood types were similar to the proportion found in other papers. The percentages of *SeLe* phenotypes varies among different regions and ethnic populations [15]. According to previous work, Se+Le+ varies by 54~78%, Se−Le+ varies by 14~31%, Se+Le− varies by 6~25%, and Se−Le− varies by 3~7% [16,24,31]. The concentrations of LNFP II and LNDFH II in Se−Le+ (a+b−) maternal milk were higher than Se+Le+ (a−b+) maternal milk, while they were absent in the milk expressed by Lewis negative subjects. 

Lactation stage, secretor status, Lewis blood type, the volume of breast milk expressed, and the province the mother came from were found to be significant factors influencing the concentrations of expressed HMOs through multiple regression model analysis in this study. Kunz et al. [19] also suggested that differences in the total amount depended on the lactation time, the secretor status, and the Lewis blood group, but that prematurity did not have an influence. Austin et al. [17] indicated that lactation stage was the most significant influencing factor of HMO concentrations, and there were no significant correlations between HMO concentrations and geographical location or mode of delivery. Some studies found there were significant differences in oligosaccharide profiles between different geographical locations [1,40,41]. These studies generally included different countries or the ethnicity of mothers [17]. The province the subjects came from also affects the concentration of total oligosaccharides, and HMO concentration showed significant differences in our study between different provinces. There are many factors that may be closely associated with the province the mothers come from, which may be affecting this, such as their dietary habits. However, there were no significant differences between rural areas and urban areas, or between coastal areas and inland areas. The geographic regional difference was only reflected in the low total HMO concentration in one province. Although human milk samples were collected from mothers from eight provinces in China, they were mainly Han ethnicity mothers and presumably have similar cultural and dietary habits, as well as a similar genetic background, which may have led to regional similarities. In works by other authors, prematurity has been shown to be significant, which was not demonstrated in this study. Wang et al. [42] concluded that there were consistently higher levels (13~23%) of oligosaccharide-bound sialic acid in preterm milk samples than in term milk samples. They had particularly low sample numbers for preterm infants, with only 21 (4.4%), so this could just be related to the small sample size leading to a sampling error. Different oligosaccharides were analyzed, which may affect the total concentrations of HMO. The percentages of *SeLe* phenotypes varies among different regions and ethnic populations, and the phenotype of *SeLe* could affect the concentrations of HMO. 

This study is not without some limitations. It is known that more than 150 HMO exist in human milk; we have analyzed 24 representative HMO and more types of HMO need to be studied. Our study population is predominantly of Han Chinese, and findings may not be applicable to other ethnic groups within the wider Chinese population. As regards the HMO concentration in mothers with premature infants, our study population contained relatively low numbers, including only 21 subjects. This may not be sufficiently powered to demonstrate differences demonstrated in other papers. The relationship between infant growth and development and oligosaccharides has not been discussed, which will be the form of future research of this group.

## 5. Conclusions

In conclusion, this is one of the largest cross-sectional studies conducted on factors affecting HMO concentrations and presents some findings in a Chinese population for the first time. HMO concentrations change throughout lactation and are influenced by the maternal secretor gene status, Lewis blood type, the volume of breast milk expressed, and the province the mother came from. There may be a mechanism for co-regulation of secretion for some oligosaccharides, such as 2′FL vs. 3FL, 2′FL vs. LNnT, and LNT. Our findings suggest that maternal BMI, maternal age, prematurity, mode of delivery, infants’ gender, maternal education level, maternal occupation, maternal allergic history, maternal anemia, pregnancy-induced hypertension, gestational diabetes, and parity were not significantly associated with total HMO concentration. 

## Figures and Tables

**Table 1:** Characteristics of the study subjects.

Characteristics	Mean ± SD or Proportion (%)
Maternal age (years)	26.8 ± 4.2 (*n* = 481)
Maternal BMI (kg/m^2^)	23.3 ± 3.5 (*n* = 481)
Infant’s gender (boy)	54.3% (*n* = 261)
Preterm	4.4% (*n* = 21)
Birth weight of term infants (g)	3491.3 ± 1060.9 (*n* = 460)
Birth weight of preterm infants (g)	2845.2 ± 544.0 (*n* = 21)
C-section rate	45.7% (*n* = 220)
Lactation stage	
Colostrum	33.7% (*n* = 162)
Transitional milk	35.3% (*n* = 170)
Mature milk	31.0% (*n* = 149)
Residential area	
Rural area	36.8% (*n* = 177)
Urban area	63.2% (*n* = 304)
Geographical region	
Coastal area	31.8% (*n* = 153)
Inland area	68.2% (*n* = 328)

## Data Availability

The datasets generated or analyzed during the current study are not publicly available due to the data management requirements of our institution, but they are available from the corresponding author on reasonable request.

## Supplementary Materials

The following supporting information can be downloaded at: https://www.mdpi.com/article/10.3390/nu15061408/s1, Table S1: Concentrations of oligosaccharides at different lactation stages. Table S2: Percentage of human milk oligosaccharides in different blood groups at the three lactation stages. Table S3: Correlation of human milk oligosaccharides.

## Abbreviations

Human milk oligosaccharides (HMOs), multi-reaction monitoring (MRM), analysis of variance (ANOVA), principal component analysis (PCA), 2’-fucosyllactose (2’-FL), lacto-N-tetraose (LNT), lacto-N-neotetraose (LNnT), sialyllacto-N-tetraose b (LSTb), sialyllacto-N-tetraose c (LSTc), lacto-N-fucopentaose I (LNFP-I), lacto-N-fucopentaose II (LNFP-II), lacto-N-fucopentaose III (LNFP-III), disialylated lacto-N-tetraose (DSLNT), difucosyl-para-lacto-N-neohexaose (DFpLNnH), difucosyl-lacto-N-hexaose (DFLNH[a]), 3-sialyllactose (3’-SL), 6-sialyllactose (6’-SL), 3-fucosyllactose (3-FL), lactodifucotetraose (LDFT), lacto-N-neo-difucohexaose I (LNnDFH-I), monofucosyl-lacto-N-neohexaose (MFLNnH), monofucosyl-lacto-N-hexaose I (MFLNH-I), monofucosyl-lacto-N-hexaose III (MFLNH-III), lacto-N-neo-difucohexaose II (LNnDFH-II), lacto-N-difucohexaose I (LNDFH-I), lacto-N-difucohexaose II (LNDFH-II), 3-sialyl-latco-N-fucopentaose II (3’-SLNFP II), 6-sialyl-latco-N-fucopentaose VI (6’-SLNFP VI).
