# Supplementary material for: Human Milk Oligosaccharides Are Associated with Lactation Stage and Lewis Phenotype in a Chinese Population
Source: Nutrients. 2023 Mar 15;15(6):1408. doi: 10.3390/nu15061408 (PMC10059825; doi:10.3390/nu15061408)
Supplement: Supplementary file 1 [file nutrients-15-01408-s001.zip › supplementary table S1 and S2.pdf]

Table S1 Concentrations of oligosaccharides at different lactation stages (mg/L)

Lactation time / Oligosaccharides	Colostrum		Transitional milk		Mature milk	
	Median	N	Median	N	Median	N
2'FL	3127.5	126	2025.0	133	1350.0	120
3-FL	261.0	160	236.3	170	580.5	147
3'SL	270.0	162	171.0	170	157.5	148
6SL	841.5	162	1141.0	169	252.0	135
LNT	1120.5	162	1062.0	170	356.1	148
LNnT	1071.0	162	486.0	169	219.9	146
LDFT	306.0	146	144.0	149	216.0	135
LNFP_I	1944.0	136	1143.0	141	360.0	113
LNFP_III	400.5	162	342.0	170	315.0	148
LNFP-II	382.5	140	513.0	161	387.0	141
DSLNT	684.0	162	414.0	170	171.0	147
LNnDFH-II	27.0	134	21.6	133	35.5	132
LNDFH_II	193.5	148	164.3	161	104.6	142
LNDFH-I&LNnDFH-I	1134.0	132	630.0	147	486.0	129
LSTb	63.0	137	99.0	158	48.1	126
LSTc	351.0	162	233.8	168	63.0	140
3'SLNFPII&6'SLNFPVI	162.0	161	126.0	169	54.0	147
MFLNnH	54.0	158	90.0	169	45.0	144
MFLNH-III	117.0	161	245.8	170	90.0	147
MFLNH-I	99.0	131	112.5	142	18.0	93
DFpLNnH	180.0	161	111.9	170	56.5	148
DFLNH(a)	396.0	161	271.0	170	130.5	148
Total	13588.9	162	10653.5	170	6048.0	148

Table S2 Percentage of human milk oligosaccharides in different blood groups at the three lactation stages.

	Colostrum				Transitional milk				Mature milk			
	se+Le+ (a-b+)	se+Le- (a-b-)	se-Le+ (a+b-)	se-Le- (a-b-)	se+Le+ (a-b+)	se+Le- (a-b-)	se-Le+ (a+b-)	se-Le- (a-b-)	se+Le+ (a-b+)	se+Le- (a-b-)	se-Le+ (a+b-)	se-Le- (a-b-)
2'-FL	20.8%	33.8%	0.6%	0.0%	18.7%	23.9%	0.5%	0.0%	20.9%	38.8%	0.9%	0.0%
LNFP_I	13.6%	20.4%	0.2%	0.9%	11.0%	25.8%	0.1%	3.4%	5.5%	11.9%	0.6%	2.7%
LNDFH-I&LNnDFH-I	9.7%	6.8%	1.0%	0.0%	7.7%	0.1%	0.7%	0.0%	9.4%	0.0%	0.5%	0.0%
LNnT	8.0%	8.2%	6.3%	10.6%	4.9%	5.8%	3.0%	0.9%	4.4%	3.6%	2.2%	3.2%
LNT	7.1%	4.2%	18.8%	39.2%	8.9%	9.5%	18.7%	11.8%	5.4%	2.9%	11.0%	55.2%
6'-SL	5.9%	5.5%	8.5%	6.2%	10.5%	9.4%	11.2%	29.1%	5.5%	8.5%	2.8%	4.7%
DSLNT	4.5%	4.0%	7.8%	9.8%	3.5%	4.9%	5.6%	7.1%	3.1%	2.2%	3.0%	2.2%
DFLNH(a)	3.1%	4.9%	2.5%	3.7%	2.6%	4.8%	2.1%	4.8%	1.9%	1.2%	2.8%	3.3%
LDFT	3.0%	0.8%	0.2%	0.0%	1.7%	0.5%	0.1%	0.1%	4.4%	1.0%	0.4%	0.7%
LNFP_III	2.6%	1.9%	6.3%	9.7%	2.7%	2.0%	4.7%	7.9%	4.7%	2.0%	7.1%	17.4%
LSTc	2.5%	2.8%	3.4%	5.2%	2.1%	3.1%	2.3%	6.4%	1.2%	0.8%	1.1%	3.0%
LNFP-II	2.2%	0.1%	17.6%	0.0%	3.5%	0.3%	21.2%	0.0%	5.3%	0.1%	19.5%	0.0%
3'-SL	1.8%	2.4%	2.9%	2.0%	1.6%	1.5%	1.6%	5.6%	2.5%	2.0%	2.7%	4.3%
3-FL	1.7%	0.4%	8.1%	1.6%	1.7%	0.8%	8.3%	3.3%	7.0%	2.4%	24.0%	8.4%
LNDFH_II	1.6%	0.0%	0.8%	0.0%	1.7%	0.1%	0.8%	0.0%	2.0%	0.0%	1.0%	0.0%
DFpLNnH	1.1%	1.3%	2.9%	1.5%	0.9%	1.0%	1.9%	1.0%	0.8%	0.4%	1.6%	1.0%
3'-SLNFPII&6'-SLNFPVI	1.0%	0.7%	3.0%	1.8%	1.0%	0.7%	3.4%	1.6%	0.8%	0.4%	1.2%	0.7%
MFLNH-III	0.7%	0.4%	2.2%	3.0%	1.9%	1.7%	4.0%	7.8%	1.2%	0.8%	2.7%	4.3%

MFLNH-I	0.7%	1.0%	0.1%	0.0%	1.1%	2.8%	0.1%	0.1%	0.4%	5.3%	0.1%	0.3%
LSTb	0.5%	0.4%	0.6%	0.5%	1.0%	0.3%	0.7%	1.3%	0.8%	0.6%	0.9%	1.2%
MFLNnH	0.4%	0.2%	1.0%	0.4%	0.8%	0.3%	1.2%	0.5%	0.7%	0.5%	0.7%	1.3%
LNnDFH-II	0.1%	0.1%	1.1%	0.2%	0.1%	0.1%	0.7%	0.6%	0.4%	0.1%	1.4%	0.3%
Total	100.0%	100.0%	100.0%	100.0%	100.0%	100.0%	100.0%	100.0%	100.0%	100.0%	100.0%	100.0%
